# Supplementary material for: Radio frequency emissions from dark-matter-candidate magnetized quark nuggets interacting with matter
Source: Sci Rep. 2020 Aug 13;10:13756. doi: 10.1038/s41598-020-70718-3 (PMC7426835; doi:10.1038/s41598-020-70718-3)
Supplement: Supplementary file 1 — Supplementary file [file 41598_2020_70718_MOESM1_ESM.pdf]

Supplementary Information for

Radio frequency emissions from dark-matter-candidate magnetized quark nuggets interacting with matter

J. Pace VanDevender*, C. Jerald Buchenauer, Chunpei Cai, Aaron P. VanDevender, and Benjamin A. Ulmen
*Corresponding author at pace@vandevender.com

Supplementary Note: Quark-nugget research summary

Witten [6] showed quark-nuggets are in the theoretically predicted, ultra-dense, color-flavor-locked (CFL) phase [17] of quark matter. Steiner, *et al.* [18] showed that the ground state of the CFL phase is color neutral and that color neutrality forces electric charge neutrality, which minimizes electromagnetic emissions. However, Xia, *et al.* [10] found that quark depletion causes the ratio Q/A of electric charge Q to baryon number A to be non-zero and varying at $Q/A \sim 0.32\, A^{-1/3}$ for $3 < A < 10^5$. In addition to this core charge, they find that there is a large surface charge and a neutralizing cloud of charge to give a net zero electric charge for sufficiently large A. So quark nuggets with $A \gg 1$ are both dark and very difficult to detect with astrophysical observations.

Witten and Xia, *et al.* also showed their density should be somewhat larger than the density of nuclei, and their mass very large, even the mass of a star. Large quark nuggets are predicted to be stable [6, 7, 17-19] with mass between 10^{-8} kg and 10^{20} kg within a plausible but uncertain range of assumed parameters of quantum chromodynamics (QCD) and the MIT bag model with its inherent limitations [20].

Although Witten assumed a first-order phase transition formed quark nuggets, Aoki, *et al.*[21] showed that the finite-temperature QCD transition that formed quark nuggets in the hot early universe was very likely an analytic crossover, involving a rapid change as the temperature varied, but not a real phase transition. Recent simulations by T. Bhattacharya, *et al*. [22] support the crossover process.

A combination of quark nuggets and anti-quark nuggets have also been proposed within constraints imposed by observations of neutrino flux [23]. Zhitnitsky [9] proposed that Axion Quark Nuggets (AQN) that forms quark and anti-quark nuggets were generated by the collapse of the axion domain wall network. Although the model relies on the hypothetical particle that is a proposed extension of the Standard Model to explain CP violation, it appears to explain a wide variety of longstanding problems and leads to quark and anti-quark nuggets with a narrow mass distribution at ~10 kg [24]. Atreya, *et al*. [25] also found that CP-violating quark and anti-quark scatterings from moving Z(3) domain walls should form quark and anti-quark nuggets, regardless of the order of the quark-hadron phase transition.

Experiments by A. Bazavov, *et al*. [26] at the Relativistic Heavy Ion Collider (RHIC) have provided the first indirect evidence of strange baryonic matter. Additional experiments at RHIC may determine whether the process is a first order phase transition or the crossover process. In either case, quark nuggets could have theoretically formed in the early universe.

In 2001, Wandelt, *et al*. [12] showed that quark nuggets meet all the theoretical requirements for dark matter and are not excluded by observations when the stopping power for quark nuggets in

the materials covering a detector is properly considered and when the average mass is >10^5 GeV (~2×10^{-22} kg). In 2014, Tulin [14] surveyed additional simulations of increasing sophistication and updated the results of Wandelt, *et al*. The combined results help establish the allowed range and velocity dependence of the strength parameter and strengthen the case for quark nuggets. In 2015, Burdin, *et al.* [27] examined all non-accelerator candidates for stable dark matter and also concluded that quark nuggets meet the requirements for dark matter and have not been excluded experimentally. Jacobs, Starkman, and Lynn [11] found that combined Earth-based, astrophysical, and cosmological observations still allow quark nuggets of mass 0.055 to 10^{14} kg and 2×10^{17} to 4×10^{21} kg to contribute substantially to dark matter. The large mass means the number per unit volume of space is small, so detecting them requires a very large-area detector.

These studies did not consider an intrinsic magnetic field within quark nuggets. However, Tatsumi [15] has shown that the lowest-energy configuration of a quark nugget depends on the QCD coupling constant and can be a ferromagnetic liquid that can account for magnetars. He calculates the value of the magnetic field at the surface of a quark-nugget core inside a magnetar to be $10^{12\pm1}$ T, which is large compared to expected values for the magnetic field at the surface of a magnetar star with a quark-nugget core. For a quark nugget of radius r_{QN} and a magnetar of radius r_s, the magnetic field scales as $(r_{QN}/r_s)^3$. Therefore, the surface magnetic field of a magnetar is smaller than 10^{12} T because $r_s > r_{QN}$. Since quark-nugget dark matter is bare, the surface magnetic field of what we wish to detect is $10^{12\pm1}$ T.

Although the cross section for interacting with dense matter is greatly enhanced [16] by the magnetic field which falls off as radius r_{QN}^{-3}, the collision cross section is still many orders of magnitude too small to violate the collision requirements [11, 12, 14, 27] for dark matter and will be discussed below.

Chakrabarty [28] showed that the stability of quark nuggets increases with increasing external magnetic field ≤ 10^{16} T, so the large self-field described by Tatsumi should enhance their stability. Ping, *et al.* [29] showed that magnetized quark nuggets should be absolutely stable with the newly-developed equivparticle model, so the large self-field described by Tatsumi should ensure that quark nuggets with sufficiently large baryon number will not decay by the weak interaction.

The large magnetic field also alters MQN interaction with ordinary matter through the greatly-enhanced stopping power of the magnetopause around high-velocity MQNs moving through a plasma [16]. Searches [30] for quark nuggets with underground detectors would not be sensitive to highly magnetized quark nuggets, which cannot penetrate the material above the detector. For example, the paper by Gorham and Rotter [23] about constraints on anti-quark nugget dark matter (which do not constrain quark-nuggets unless the ratio of anti-quark nuggets to quark nuggets is shown to be large) assumes that limits on the flux of magnetic monopoles from analysis by Price, *et al.* [31] of geologic mica buried under 3 km of rock are also applicable to quark nuggets. Gorham and Rotter also cite work by Porter, *et al.* [32-33] as constraining quark-nugget (nuclearite) contributions to dark matter by the absence of meteor-like objects in the lower atmosphere that are fast enough to be quark nuggets. Bassan, *et al*. [34] looked for quark nuggets (nuclearites) with gravitational wave detectors and found signals much less than expected for the flux of dark matter. However, all of these analyses assumed quark nuggets can reach the detector volume because the cross section for momentum transfer is the geometric

cross section. In contrast, the MQN magnetopause cross section [16] is many orders of magnitude larger and prevents all but the most massive MQNs from being detected.

Supplementary Results: Representative Data Tables for Sensor Design

Table S1 through Table S4 respectively provide representative events for B_o equals 1.5×10^{12} T, 2.0×10^{12} T, 2.5×10^{12} T, and 3.0×10^{12} T. The tables show the computed parameters for MQN events with RF power greater than 1 nW and with sufficient flux to place them in the most probable 80% of the total number of events. The information should be useful for designing sensors for detecting MQNs.

The computed mass distribution [4] of MQNs extend over 30 orders of magnitude, from ~ 10^{-24} kg to > 10^{6} kg. To cover such a large range, we approximated the mass distributions by the distribution of decadal masses. Calculated trajectories for the masses in the logarithmic center of each decade of mass approximate the behaviors of all the MQNs in that decade. Decade mass is listed in the left most column of the tables. The total flux for all masses in a decade of mass have been calculated in aggregation simulations [4] and that decadal flux is given in the 7th column of the Tables. Multiplying that flux by the cross section corresponding to the closest-approach altitude h and extent Δh for the entry and the 5.56 factor to generalize the unidirectional result to omni-directional results gives the event rate in the 8th column. The spin-down time is shown in the 5th column. Each of the four tables is for the indicated value of the surface-magnetic-field parameter B_o.

Table S1: Representative results for $B_o = 1.5 \times 10^{12}$ T.

MQN Mass (kg)	Altitude h (m)	Delta_h (m)	Frequency (Hz)	τ _down (s)	RF power (W)	Flux (n/m^2/y/sr)	Number/y for all directions
3.00×10^{-8}	1.03×10^{5}	1.03×10^{4}	2.61×10^{8}	5.97×10^{1}	1.00×10^{-9}	2.12×10^{-13}	3.16×10^{3}
3.00×10^{-8}	9.28×10^{4}	9.28×10^{3}	3.77×10^{8}	2.86×10^{1}	4.38×10^{-9}	2.12×10^{-13}	2.58×10^{3}
3.00×10^{-8}	8.36×10^{4}	8.36×10^{3}	4.89×10^{8}	1.70×10^{1}	1.23×10^{-8}	2.12×10^{-13}	2.12×10^{3}
3.00×10^{-5}	9.28×10^{4}	9.28×10^{3}	4.06×10^{7}	2.47×10^{2}	5.88×10^{-7}	1.20×10^{-13}	1.79×10^{3}
3.00×10^{-6}	1.03×10^{5}	1.03×10^{4}	5.81×10^{7}	2.60×10^{2}	2.47×10^{-8}	1.16×10^{-13}	1.73×10^{3}
3.00×10^{-5}	8.36×10^{4}	8.36×10^{3}	5.87×10^{7}	1.18×10^{2}	2.57×10^{-6}	1.20×10^{-13}	1.47×10^{3}
3.00×10^{-6}	9.28×10^{4}	9.28×10^{3}	8.65×10^{7}	1.17×10^{2}	1.21×10^{-7}	1.16×10^{-13}	1.41×10^{3}
3.00×10^{-5}	7.52×10^{4}	7.52×10^{3}	7.99×10^{7}	6.38×10^{1}	8.81×10^{-6}	1.20×10^{-13}	1.20×10^{3}
3.00×10^{-6}	8.36×10^{4}	8.36×10^{3}	1.23×10^{8}	5.84×10^{1}	4.88×10^{-7}	1.16×10^{-13}	1.16×10^{3}
3.00×10^{-7}	1.03×10^{5}	1.03×10^{4}	1.24×10^{8}	1.23×10^{2}	5.08×10^{-9}	7.40×10^{-14}	1.10×10^{3}
3.00×10^{-5}	6.77×10^{4}	6.77×10^{3}	1.03×10^{8}	3.80×10^{1}	2.48×10^{-5}	1.20×10^{-13}	9.85×10^{2}
3.00×10^{-6}	7.52×10^{4}	7.52×10^{3}	1.64×10^{8}	3.28×10^{1}	1.55×10^{-6}	1.16×10^{-13}	9.51×10^{2}
3.00×10^{-7}	9.28×10^{4}	9.28×10^{3}	1.82×10^{8}	5.67×10^{1}	2.40×10^{-8}	7.40×10^{-14}	9.04×10^{2}
3.00×10^{-5}	6.09×10^{4}	6.09×10^{3}	1.26×10^{8}	2.55×10^{1}	5.50×10^{-5}	1.20×10^{-13}	8.09×10^{2}
3.00×10^{-6}	6.77×10^{4}	6.77×10^{3}	2.02×10^{8}	2.16×10^{1}	3.57×10^{-6}	1.16×10^{-13}	7.81×10^{2}
3.00×10^{-7}	8.36×10^{4}	8.36×10^{3}	2.51×10^{8}	3.00×10^{1}	8.57×10^{-8}	7.40×10^{-14}	7.41×10^{2}
3.00×10^{-3}	7.52×10^{4}	7.52×10^{3}	1.78×10^{7}	2.77×10^{2}	2.16×10^{-4}	4.46×10^{-14}	6.65×10^{2}
3.00×10^{-7}	7.52×10^{4}	7.52×10^{3}	3.15×10^{8}	1.90×10^{1}	2.14×10^{-7}	7.40×10^{-14}	6.08×10^{2}
3.00×10^{-3}	6.77×10^{4}	6.77×10^{3}	2.40×10^{7}	1.53×10^{2}	7.14×10^{-4}	4.46×10^{-14}	5.44×10^{2}
3.00×10^{-4}	8.36×10^{4}	8.36×10^{3}	2.73×10^{7}	2.54×10^{2}	1.20×10^{-5}	3.51×10^{-14}	5.23×10^{2}
3.00×10^{-3}	6.09×10^{4}	6.09×10^{3}	3.07×10^{7}	9.28×10^{1}	1.93×10^{-3}	4.46×10^{-14}	4.46×10^{2}
3.00×10^{-4}	7.52×10^{4}	7.52×10^{3}	3.79×10^{7}	1.32×10^{2}	4.45×10^{-5}	3.51×10^{-14}	4.29×10^{2}
3.00×10^{-1}	4.93×10^{4}	4.93×10^{3}	1.07×10^{7}	1.65×10^{2}	2.84×10^{-1}	2.63×10^{-14}	3.93×10^{2}
3.00×10^{-3}	5.48×10^{4}	5.48×10^{3}	3.83×10^{7}	5.99×10^{1}	4.63×10^{-3}	4.46×10^{-14}	3.66×10^{2}
3.00×10^{-4}	6.77×10^{4}	6.77×10^{3}	5.06×10^{7}	7.39×10^{1}	1.41×10^{-4}	3.51×10^{-14}	3.51×10^{2}
3.00×10^{-1}	4.44×10^{4}	4.44×10^{3}	1.29×10^{7}	1.13×10^{2}	6.05×10^{-1}	2.63×10^{-14}	3.21×10^{2}
3.00×10^{-3}	4.93×10^{4}	4.93×10^{3}	4.58×10^{7}	4.19×10^{1}	9.49×10^{-3}	4.46×10^{-14}	3.00×10^{2}
3.00×10^{-4}	6.09×10^{4}	6.09×10^{3}	6.36×10^{7}	4.68×10^{1}	3.53×10^{-4}	3.51×10^{-14}	2.88×10^{2}
3.00×10^{-2}	6.09×10^{4}	6.09×10^{3}	1.46×10^{7}	1.92×10^{2}	9.75×10^{-3}	1.93×10^{-14}	2.88×10^{2}
3.00	3.60×10^{4}	3.60×10^{3}	8.43×10^{6}	1.23×10^{2}	1.09×10^{1}	1.93×10^{-14}	2.88×10^{2}
3.00×10^{-1}	4.00×10^{4}	4.00×10^{3}	1.52×10^{7}	8.21×10^{1}	1.14	2.63×10^{-14}	2.63×10^{2}
3.00×10^{-3}	4.44×10^{4}	4.44×10^{3}	5.31×10^{7}	3.11×10^{1}	1.72×10^{-2}	4.46×10^{-14}	2.47×10^{2}
3.00×10^{-4}	5.48×10^{4}	5.48×10^{3}	7.68×10^{7}	3.20×10^{1}	7.51×10^{-4}	3.51×10^{-14}	2.37×10^{2}
3.00×10^{-2}	5.48×10^{4}	5.48×10^{3}	1.85×10^{7}	1.19×10^{2}	2.54×10^{-2}	1.93×10^{-14}	2.36×10^{2}

Table S1: Representative results for $B_o = 2.0 \times 10^{12}$ T.

MQN Mass (kg)	Altitude *h* (m)	Δ*h* (m)	Frequency (Hz)	τ _down (s)	RF power (W)	Flux ($m^{-2}y^{-1}sr^{-1}$)	Number/y for all directions
3.00×10^{6}	6.14×10^{3}	6.14×10^{2}	2.81×10^{5}	6.27×10^{2}	2.38×10^{7}	1.46×10^{-16}	1.06×10^{-1}
3.00×10^{6}	7.14×10^{3}	7.14×10^{2}	2.70×10^{5}	6.79×10^{2}	2.03×10^{7}	1.46×10^{-16}	1.06×10^{-1}
3.00×10^{6}	8.14×10^{3}	8.14×10^{2}	2.59×10^{5}	7.35×10^{2}	1.73×10^{7}	1.46×10^{-16}	1.06×10^{-1}
3.00×10^{6}	9.14×10^{3}	9.14×10^{2}	2.49×10^{5}	7.96×10^{2}	1.48×10^{7}	1.46×10^{-16}	1.06×10^{-1}
3.00×10^{6}	1.02×10^{4}	1.02×10^{3}	2.39×10^{5}	8.62×10^{2}	1.26×10^{7}	1.46×10^{-16}	1.06×10^{-1}
3.00×10^{6}	1.13×10^{4}	1.13×10^{3}	2.28×10^{5}	9.52×10^{2}	1.03×10^{7}	1.46×10^{-16}	1.06×10^{-1}
3.00×10^{6}	1.25×10^{4}	1.25×10^{3}	2.17×10^{5}	1.05×10^{3}	8.46×10^{6}	1.46×10^{-16}	1.06×10^{-1}
3.00×10^{6}	1.39×10^{4}	1.39×10^{3}	2.06×10^{5}	1.16×10^{3}	6.93×10^{6}	1.46×10^{-16}	1.06×10^{-1}
3.00×10^{6}	1.55×10^{4}	1.55×10^{3}	1.94×10^{5}	1.31×10^{3}	5.46×10^{6}	1.46×10^{-16}	1.06×10^{-1}
3.00×10^{6}	1.72×10^{4}	1.72×10^{3}	1.81×10^{5}	1.50×10^{3}	4.13×10^{6}	1.46×10^{-16}	1.06×10^{-1}
3.00×10^{6}	1.91×10^{4}	1.91×10^{3}	1.67×10^{5}	1.76×10^{3}	3.01×10^{6}	1.46×10^{-16}	1.06×10^{-1}
3.00×10^{6}	2.12×10^{4}	2.12×10^{3}	1.53×10^{5}	2.11×10^{3}	2.10×10^{6}	1.46×10^{-16}	1.06×10^{-1}
3.00×10^{6}	2.36×10^{4}	2.36×10^{3}	1.40×10^{5}	2.52×10^{3}	1.47×10^{6}	1.46×10^{-16}	1.06×10^{-1}
3.00×10^{6}	2.62×10^{4}	2.62×10^{3}	1.25×10^{5}	3.14×10^{3}	9.48×10^{5}	1.46×10^{-16}	1.06×10^{-1}
3.00×10^{6}	2.91×10^{4}	2.91×10^{3}	1.12×10^{5}	3.91×10^{3}	6.12×10^{5}	1.46×10^{-16}	1.06×10^{-1}
3.00×10^{6}	3.24×10^{4}	3.24×10^{3}	9.77×10^{4}	5.17×10^{3}	3.50×10^{5}	1.46×10^{-16}	1.06×10^{-1}
3.00×10^{6}	3.60×10^{4}	3.60×10^{3}	8.50×10^{4}	6.83×10^{3}	2.01×10^{5}	1.46×10^{-16}	1.06×10^{-1}
3.00×10^{6}	4.00×10^{4}	4.00×10^{3}	7.25×10^{4}	9.39×10^{3}	1.06×10^{5}	1.46×10^{-16}	1.06×10^{-1}
3.00×10^{6}	4.44×10^{4}	4.44×10^{3}	6.06×10^{4}	1.34×10^{4}	5.19×10^{4}	1.46×10^{-16}	1.06×10^{-1}
3.00×10^{6}	4.93×10^{4}	4.93×10^{3}	4.97×10^{4}	2.00×10^{4}	2.34×10^{4}	1.46×10^{-16}	1.06×10^{-1}
3.00×10^{6}	5.48×10^{4}	5.48×10^{3}	3.99×10^{4}	3.10×10^{4}	9.75×10^{3}	1.46×10^{-16}	1.06×10^{-1}
3.00×10^{6}	6.09×10^{4}	6.09×10^{3}	3.11×10^{4}	5.09×10^{4}	3.60×10^{3}	1.46×10^{-16}	1.06×10^{-1}
3.00×10^{6}	6.77×10^{4}	6.77×10^{3}	2.38×10^{4}	8.72×10^{4}	1.23×10^{3}	1.46×10^{-16}	1.06×10^{-1}
3.00×10^{6}	7.52×10^{4}	7.52×10^{3}	1.75×10^{4}	1.62×10^{5}	3.58×10^{2}	1.46×10^{-16}	1.06×10^{-1}
3.00×10^{6}	8.36×10^{4}	8.36×10^{3}	1.26×10^{4}	3.12×10^{5}	9.63×10^{1}	1.46×10^{-16}	1.06×10^{-1}
3.00×10^{6}	9.28×10^{4}	9.28×10^{3}	8.62×10^{3}	6.64×10^{5}	2.12×10^{1}	1.46×10^{-16}	1.06×10^{-1}
3.00×10^{6}	1.03×10^{5}	1.03×10^{4}	5.68×10^{3}	1.53×10^{6}	3.99	1.46×10^{-16}	1.06×10^{-1}
3.00×10^{6}	1.15×10^{5}	1.15×10^{4}	3.59×10^{3}	3.82×10^{6}	6.39×10^{-1}	1.46×10^{-16}	1.06×10^{-1}
3.00×10^{6}	1.27×10^{5}	1.27×10^{4}	2.16×10^{3}	1.06×10^{7}	8.40×10^{-2}	1.46×10^{-16}	1.06×10^{-1}
3.00×10^{6}	1.41×10^{5}	1.41×10^{4}	1.21×10^{3}	3.35×10^{7}	8.35×10^{-3}	1.46×10^{-16}	1.06×10^{-1}
3.00×10^{6}	1.57×10^{5}	1.57×10^{4}	6.49×10^{2}	1.17×10^{8}	6.80×10^{-4}	1.46×10^{-16}	1.06×10^{-1}
3.00×10^{6}	1.75×10^{5}	1.75×10^{4}	3.20×10^{2}	4.82×10^{8}	4.03×10^{-5}	1.46×10^{-16}	1.06×10^{-1}
3.00×10^{6}	1.94×10^{5}	1.94×10^{4}	1.47×10^{2}	2.27×10^{9}	1.81×10^{-6}	1.46×10^{-16}	1.06×10^{-1}
3.00×10^{6}	2.16×10^{5}	2.16×10^{4}	6.13×10^{1}	1.31×10^{10}	5.44×10^{-8}	1.46×10^{-16}	1.06×10^{-1}

Table S3: Representative results for B_o = 2.5 × 10^{12} T.

MQN Mass (kg)	Altitude h (m)	Δh (m)	Frequency (Hz)	τ _down (s)	RF power (W)	Flux ($m^{-2}y^{-1}sr^{-1}$)	Number/y for all directions
3.00×10^{6}	6.14×10^{3}	6.14×10^{2}	2.75×10^{5}	4.17×10^{2}	3.44×10^{7}	7.27×10^{-19}	5.29×10^{-4}
3.00×10^{6}	7.14×10^{3}	7.14×10^{2}	2.64×10^{5}	4.52×10^{2}	2.93×10^{7}	7.27×10^{-19}	5.29×10^{-4}
3.00×10^{6}	8.14×10^{3}	8.14×10^{2}	2.54×10^{5}	4.89×10^{2}	2.50×10^{7}	7.27×10^{-19}	5.29×10^{-4}
3.00×10^{6}	9.14×10^{3}	9.14×10^{2}	2.44×10^{5}	5.30×10^{2}	2.13×10^{7}	7.27×10^{-19}	5.29×10^{-4}
3.00×10^{6}	1.02×10^{4}	1.02×10^{3}	2.35×10^{5}	5.74×10^{2}	1.82×10^{7}	7.27×10^{-19}	5.29×10^{-4}
3.00×10^{6}	1.13×10^{4}	1.13×10^{3}	2.23×10^{5}	6.34×10^{2}	1.49×10^{7}	7.27×10^{-19}	5.29×10^{-4}
3.00×10^{6}	1.25×10^{4}	1.25×10^{3}	2.12×10^{5}	7.00×10^{2}	1.22×10^{7}	7.27×10^{-19}	5.29×10^{-4}
3.00×10^{6}	1.39×10^{4}	1.39×10^{3}	2.02×10^{5}	7.73×10^{2}	1.00×10^{7}	7.27×10^{-19}	5.29×10^{-4}
3.00×10^{6}	1.55×10^{4}	1.55×10^{3}	1.90×10^{5}	8.72×10^{2}	7.88×10^{6}	7.27×10^{-19}	5.29×10^{-4}
3.00×10^{6}	1.72×10^{4}	1.72×10^{3}	1.77×10^{5}	1.00×10^{3}	5.96×10^{6}	7.27×10^{-19}	5.29×10^{-4}
3.00×10^{6}	1.91×10^{4}	1.91×10^{3}	1.64×10^{5}	1.17×10^{3}	4.34×10^{6}	7.27×10^{-19}	5.29×10^{-4}
3.00×10^{6}	2.12×10^{4}	2.12×10^{3}	1.51×10^{5}	1.38×10^{3}	3.15×10^{6}	7.27×10^{-19}	5.29×10^{-4}
3.00×10^{6}	2.36×10^{4}	2.36×10^{3}	1.37×10^{5}	1.68×10^{3}	2.12×10^{6}	7.27×10^{-19}	5.29×10^{-4}
3.00×10^{6}	2.62×10^{4}	2.62×10^{3}	1.23×10^{5}	2.09×10^{3}	1.37×10^{6}	7.27×10^{-19}	5.29×10^{-4}
3.00×10^{6}	2.91×10^{4}	2.91×10^{3}	1.10×10^{5}	2.60×10^{3}	8.83×10^{5}	7.27×10^{-19}	5.29×10^{-4}
3.00×10^{6}	3.24×10^{4}	3.24×10^{3}	9.67×10^{4}	3.37×10^{3}	5.26×10^{5}	7.27×10^{-19}	5.29×10^{-4}
3.00×10^{6}	3.60×10^{4}	3.60×10^{3}	8.33×10^{4}	4.55×10^{3}	2.90×10^{5}	7.27×10^{-19}	5.29×10^{-4}
3.00×10^{6}	4.00×10^{4}	4.00×10^{3}	7.11×10^{4}	6.25×10^{3}	1.53×10^{5}	7.27×10^{-19}	5.29×10^{-4}
3.00×10^{6}	4.44×10^{4}	4.44×10^{3}	5.94×10^{4}	8.94×10^{3}	7.48×10^{4}	7.27×10^{-19}	5.29×10^{-4}
3.00×10^{6}	4.93×10^{4}	4.93×10^{3}	4.87×10^{4}	1.33×10^{4}	3.38×10^{4}	7.27×10^{-19}	5.29×10^{-4}
3.00×10^{6}	5.48×10^{4}	5.48×10^{3}	3.91×10^{4}	2.06×10^{4}	1.41×10^{4}	7.27×10^{-19}	5.29×10^{-4}
3.00×10^{6}	6.09×10^{4}	6.09×10^{3}	3.05×10^{4}	3.39×10^{4}	5.20×10^{3}	7.27×10^{-19}	5.29×10^{-4}
3.00×10^{6}	6.77×10^{4}	6.77×10^{3}	2.33×10^{4}	5.81×10^{4}	1.77×10^{3}	7.27×10^{-19}	5.29×10^{-4}
3.00×10^{6}	7.52×10^{4}	7.52×10^{3}	1.73×10^{4}	1.05×10^{5}	5.38×10^{2}	7.27×10^{-19}	5.29×10^{-4}
3.00×10^{6}	8.36×10^{4}	8.36×10^{3}	1.23×10^{4}	2.08×10^{5}	1.39×10^{2}	7.27×10^{-19}	5.29×10^{-4}
3.00×10^{6}	9.28×10^{4}	9.28×10^{3}	8.45×10^{3}	4.42×10^{5}	3.06×10^{1}	7.27×10^{-19}	5.29×10^{-4}
3.00×10^{6}	1.03×10^{5}	1.03×10^{4}	5.56×10^{3}	1.02×10^{6}	5.76	7.27×10^{-19}	5.29×10^{-4}
3.00×10^{6}	1.15×10^{5}	1.15×10^{4}	3.52×10^{3}	2.55×10^{6}	9.22×10^{-1}	7.27×10^{-19}	5.29×10^{-4}
3.00×10^{6}	1.27×10^{5}	1.27×10^{4}	2.12×10^{3}	7.03×10^{6}	1.21×10^{-1}	7.27×10^{-19}	5.29×10^{-4}
3.00×10^{6}	1.41×10^{5}	1.41×10^{4}	1.19×10^{3}	2.23×10^{7}	1.20×10^{-2}	7.27×10^{-19}	5.29×10^{-4}
3.00×10^{6}	1.57×10^{5}	1.57×10^{4}	6.36×10^{2}	7.81×10^{7}	9.81×10^{-4}	7.27×10^{-19}	5.29×10^{-4}
3.00×10^{6}	1.75×10^{5}	1.75×10^{4}	3.14×10^{2}	3.21×10^{8}	5.82×10^{-5}	7.27×10^{-19}	5.29×10^{-4}
3.00×10^{6}	1.94×10^{5}	1.94×10^{4}	1.44×10^{2}	1.51×10^{9}	2.61×10^{-6}	7.27×10^{-19}	5.29×10^{-4}
3.00×10^{6}	2.16×10^{5}	2.16×10^{4}	6.07×10^{1}	8.56×10^{9}	8.17×10^{-8}	7.27×10^{-19}	5.29×10^{-4}

Table S4: Representative results for B_o = 3.0 × 10^{12} T.

MQN Mass (kg)	Altitude h (m)	Δh (m)	Frequency (Hz)	τ _down (s)	RF power (W)	Flux ($m^{-2}y^{-1}sr^{-1}$)	Number/y for all directions
3.00	1.41×10^{5}	1.41×10^{4}	1.18×10^{5}	1.57×10^{5}	1.68×10^{-6}	9.55×10^{-18}	6.60×10^{-5}
3.00	1.27×10^{5}	1.27×10^{4}	2.08×10^{5}	5.06×10^{4}	1.62×10^{-5}	9.55×10^{-18}	6.57×10^{-5}
3.00	1.15×10^{5}	1.15×10^{4}	3.46×10^{5}	1.83×10^{4}	1.24×10^{-4}	9.55×10^{-18}	5.89×10^{-5}
3.00	1.03×10^{5}	1.03×10^{4}	5.52×10^{5}	7.20×10^{3}	8.02×10^{-4}	9.55×10^{-18}	5.28×10^{-5}
3.00×10^{-3}	7.52×10^{4}	7.52×10^{3}	1.68×10^{7}	7.81×10^{1}	6.81×10^{-4}	1.80×10^{-17}	5.06×10^{-5}
3.00	9.28×10^{4}	9.28×10^{3}	8.30×10^{5}	3.18×10^{3}	4.10×10^{-3}	9.55×10^{-18}	4.74×10^{-5}
3.00×10^{-3}	6.77×10^{4}	6.77×10^{3}	2.24×10^{7}	4.39×10^{1}	2.16×10^{-3}	1.80×10^{-17}	4.69×10^{-5}
3.00×10^{-1}	9.28×10^{4}	9.28×10^{3}	1.80×10^{6}	1.45×10^{3}	9.15×10^{-4}	9.69×10^{-18}	4.31×10^{-5}
3.00	8.36×10^{4}	8.36×10^{3}	1.21×10^{6}	1.49×10^{3}	1.86×10^{-2}	9.55×10^{-18}	4.25×10^{-5}
3.00×10^{-3}	6.09×10^{4}	6.09×10^{3}	2.87×10^{7}	2.67×10^{1}	5.84×10^{-3}	1.80×10^{-17}	4.21×10^{-5}
3.00×10^{3}	2.16×10^{5}	2.16×10^{4}	5.99×10^{2}	6.11×10^{8}	1.11×10^{-9}	2.39×10^{-18}	4.03×10^{-5}
3.00×10^{-1}	8.36×10^{4}	8.36×10^{3}	2.61×10^{6}	6.95×10^{2}	3.99×10^{-3}	9.69×10^{-18}	3.87×10^{-5}
3.00	7.52×10^{4}	7.52×10^{3}	1.70×10^{6}	7.60×10^{2}	7.20×10^{-2}	9.55×10^{-18}	3.82×10^{-5}
3.00×10^{-3}	5.48×10^{4}	5.48×10^{3}	3.50×10^{7}	1.79×10^{1}	1.30×10^{-2}	1.80×10^{-17}	3.78×10^{-5}
3.00×10^{-2}	8.36×10^{4}	8.36×10^{3}	5.61×10^{6}	3.24×10^{2}	8.55×10^{-4}	1.01×10^{-17}	3.62×10^{-5}
3.00×10^{3}	1.94×10^{5}	1.94×10^{4}	1.42×10^{3}	1.08×10^{8}	3.55×10^{-8}	2.39×10^{-18}	3.59×10^{-5}
3.00×10^{-1}	7.52×10^{4}	7.52×10^{3}	3.65×10^{6}	3.53×10^{2}	1.54×10^{-2}	9.69×10^{-18}	3.47×10^{-5}
3.00×10^{-4}	6.09×10^{4}	6.09×10^{3}	5.75×10^{7}	1.43×10^{1}	9.48×10^{-4}	1.49×10^{-17}	3.47×10^{-5}
3.00	6.77×10^{4}	6.77×10^{3}	2.29×10^{6}	4.18×10^{2}	2.38×10^{-1}	9.55×10^{-18}	3.42×10^{-5}
3.00×10^{-3}	4.93×10^{4}	4.93×10^{3}	4.10×10^{7}	1.30×10^{1}	2.45×10^{-2}	1.80×10^{-17}	3.40×10^{-5}
3.00×10^{-2}	7.52×10^{4}	7.52×10^{3}	7.79×10^{6}	1.68×10^{2}	3.18×10^{-3}	1.01×10^{-17}	3.25×10^{-5}
3.00×10^{3}	1.75×10^{5}	1.75×10^{4}	3.09×10^{3}	2.29×10^{7}	7.92×10^{-7}	2.39×10^{-18}	3.21×10^{-5}
3.00×10^{-1}	6.77×10^{4}	6.77×10^{3}	4.93×10^{6}	1.95×10^{2}	5.10×10^{-2}	9.69×10^{-18}	3.12×10^{-5}
3.00×10^{-4}	5.48×10^{4}	5.48×10^{3}	6.75×10^{7}	1.04×10^{1}	1.79×10^{-3}	1.49×10^{-17}	3.12×10^{-5}
3.00	6.09×10^{4}	6.09×10^{3}	3.00×10^{6}	2.44×10^{2}	6.96×10^{-1}	9.55×10^{-18}	3.08×10^{-5}
3.00×10^{-2}	6.77×10^{4}	6.77×10^{3}	1.05×10^{7}	9.24×10^{1}	1.05×10^{-2}	1.01×10^{-17}	2.92×10^{-5}
3.00×10^{3}	1.57×10^{5}	1.57×10^{4}	6.27×10^{3}	5.58×10^{6}	1.34×10^{-5}	2.39×10^{-18}	2.87×10^{-5}
3.00×10^{-1}	6.09×10^{4}	6.09×10^{3}	6.44×10^{6}	1.14×10^{2}	1.49×10^{-1}	9.69×10^{-18}	2.80×10^{-5}
3.00×10^{1}	1.57×10^{5}	1.57×10^{4}	2.90×10^{4}	1.21×10^{6}	6.14×10^{-7}	2.92×10^{-18}	2.80×10^{-5}
3.00	5.48×10^{4}	5.48×10^{3}	3.80×10^{6}	1.52×10^{2}	1.81	9.55×10^{-18}	2.76×10^{-5}
3.00×10^{-2}	6.09×10^{4}	6.09×10^{3}	1.36×10^{7}	5.51×10^{1}	2.95×10^{-2}	1.01×10^{-17}	2.62×10^{-5}
3.00×10^{3}	1.41×10^{5}	1.41×10^{4}	1.17×10^{4}	1.59×10^{6}	1.64×10^{-4}	2.39×10^{-18}	2.56×10^{-5}
3.00×10^{-1}	5.48×10^{4}	5.48×10^{3}	8.10×10^{6}	7.19×10^{1}	3.73×10^{-1}	9.69×10^{-18}	2.52×10^{-5}
3.00×10^{1}	1.41×10^{5}	1.41×10^{4}	5.49×10^{4}	3.38×10^{5}	7.84×10^{-6}	2.92×10^{-18}	2.50×10^{-5}
